# Are Confident Parents Really Aware of Children’s Online Risks? A Conceptual Model and Validation of Parental Self-Efficacy, Mediation, and Awareness Scales

**DOI:** 10.1007/s42380-023-00157-x

**Published:** 2023-02-08

**Authors:** Seffetullah Kuldas, Aikaterini Sargioti, Elisabeth Staksrud, Darran Heaney, James O’Higgins Norman

**Affiliations:** 1 Department of Media and Communication, University of Oslo, Oslo, Norway; 2 DCU Anti-Bullying Centre, Institute of Education, Dublin City University, Dublin, Ireland

**Keywords:** Parental self-efficacy, Parental mediation, Parental awareness, Online risk, Online safety, Internet use

## Abstract

Children’s use of the Internet comes with both risks and opportunities. To minimize risks and maximize opportunities, parents may choose to observe, enable, and/or restrict their children’s Internet use. However, parents’ high confidence in their children’s online safety can itself be a risk factor inhibiting parental awareness of online risks. This research aims to test whether confident parents are accurately aware of how frequently their child has experienced risks online. To this end, construct validity and reliability of scales measuring parental self-efficacy, restrictive-enabling-observant mediation, awareness, and Internet use were established first. Next, a conceptual model of parental awareness was proposed. These results were based on a two-parameter-logistic-model of item response theory, minimum-rank factor analysis, and parallel-mediation analysis of self-reports by a convenience sample of 388 parents in Ireland (Autumn 2019). Confident parents mostly reported their child experienced no online risk in the past couple of months, whereas unconfident parents reported their child experienced an online risk once, twice, or more times. Results of the mediation analysis indicated that confident parents likely underestimated, whereas unconfident parents overestimated, how frequently their child experienced an online risk. The accuracy of parental awareness depended on their mediation strategies, particularly restrictive mediation. Further research is needed to test whether training parents on self-efficacy and mediation of children’s Internet use raises their awareness of the children’s online risks.

Research over the last two decades has reached no conceptual clarity on how parental self-efficacy, mediation, and awareness affect each other when parenting for children’s Internet use (Kuldas et al., 2023). The lack of clarity especially pertains to: (a) whether parents set rules to become aware or after they become aware of their children’s online risk experiences, and (b) whether parental self-efficacy is a *result* or *antecedent* of parental mediation (Kuldas et al., 2023). A recent review of these issues (Kuldas et al., 2023) found also no definitional consensus on the three main concepts, but an increasing use of the following definitions:

First, *parental self-efficacy* refers to Internet/digital parenting *confidence* in their own promotive-protective skills and efforts, such as digital literacy, open parent–child communication, and rule-setting. Second, *parental mediation* is defined as promotive-protective strategies that parents use to *restrict*, *enable*, and/or *observe* their children’s Internet use, including child-initiated communications about privacy, activity, conduct, contact, content, or time spent online. Third, *parental awareness* refers to the extent to which parents accurately estimate or know how frequently their children have experienced risks online. These parental factors have a twofold aim: (a) to ensure children’s online safety and access to online opportunities (e.g., knowledge acquisition, identity development, entertainment, or social interaction) as well as (b) to develop children’s own ability and willingness to use online opportunities and avoid/tackle online risks (Kuldas et al., 2023).

The aim of achievement can be either facilitated or hindered by the interplays among parental self-efficacy, mediation, and awareness. In a series of studies over the past decade, parents who self-reported high confidence in their children’s online safety inaccurately estimated the frequency of their children’s online risk experiences (Byrne et al., 2014; Caivano, et al., 2020; Dehue et al., 2008; McGuire & O’Higgins Norman, 2017; Livingstone et al., 2011; National Advisory Council for Online Safety [NACOS], 2021; O’Neill et al., 2011; Symons et al., 2017). This lack of awareness can prevent parents from tailoring their mediation strategies to their children’s needs for online safety and opportunities. However, the extent to which lack of awareness among parents is related to their mediation strategies and self-efficacy levels has yet to be tested. This requires an accurate measurement and conceptual framework of the parental factors that increase or decrease their awareness (Caivano et al., 2020; Symons et al., 2017). Therefore, the present research tests and proposes a conceptual framework and measurement scales for parental mediation, self-efficacy, and awareness of children’s online risks.

Empirical research on parental awareness is scarce, mainly because earlier studies focused on parental mediation strategies in terms of prevention rather than awareness of online risks (Caivano et al., 2020; Symons et al., 2017). There are only a few studies on restrictive and enabling mediation in relation to parental awareness of children as perpetrators (Barlett & Fennel, 2018; Dehue et al., 2008) or victims of cyberbullying behavior (Dehue et al., 2008; Symons et al., 2017). More recently, Caivano et al. (2020) focused only on restrictive mediation in relation to parental awareness of children’s experiences of cyberbullying as a perpetrator, victim, or bystander. No conclusion was reached on which parental mediation strategy best predicts parental awareness.

The most common method for estimating parental awareness is a statistical comparison between parents and children’s self-reports (Symons et al., 2017). However, children tend to overestimate how frequently they experience online risks (Byrne et al., 2014; Caivano et al., 2020; Dehue et al., 2008; Symons et al., 2017). Given that both parents and children inaccurately estimate the frequency, children’s self-reports can be an inaccurate reference for substantiating parents’ self-reports, or vice versa.

The present research argues that a criterion-based assessment, as an alternative method to the norm-based assessment (i.e., parent–child comparison), can be used for an accurate estimation of risk awareness. One criterion reference is parents’ self-reports of mediation strategies for children’s Internet use; it allows researchers to test if what parents do to become aware of online risks is evidence for what they know how frequently their children have experienced risks online (i.e., testing whether the inaccurate estimation among parents with higher self-efficacy is due to their mediation strategies). To test this argument, relationships between parental self-efficacy, mediation, and awareness have yet to be conceptualized and measured. Therefore, the present research is focused on facilitating the conceptualization and measurement by proposing a conceptual model of parental awareness after testing the measurement model, the construct validity and reliability of scales measuring (a) parental mediation of children’s Internet use (i.e., testing the restrictive-enabling-observant mediation trichotomy), (b) parental awareness of children’s online risks, (c) parental self-efficacy in their child’s online safety, and (d) parental Internet use.

## Literature Review

### Parental Awareness of Children’s Online Risks

Parenting for children’s Internet use has changed dramatically, especially with the use of social media for self-presentation, including online activities related to their privacy, intimacy, and sexuality (Kuldas et al., 2021). Although every online risk is not necessarily harmful or deliberate, children can experience online *content*, *contact*, *and conduct risks*, which can be exemplified with a violation of personal *privacy* (Staksrud & Livingstone, 2009). First, content risks can be illegal, pornographic, violent, racist, hateful, or misinformative videos, comments, images, and/or texts; but increasingly worrisome are those stimulating self-harm (Livingstone, 2019; Livingstone & Haddon, 2008; Livingstone & Helsper, 2010; Staksrud & Livingstone, 2009). Second, contact risks are online communications that include cyberbullying with hurtful messages (Livingstone, 2019), non-consensual sharing of intimate images (Livingstone et al., 2017), or grooming (NACOS, 2021; Staksrud, 2013). Third, conduct risks refer to misconduct behaviors online, such as cyberbullying, non-consensual sexting, or gambling (Livingstone et al., 2017). In general, parents are unaware (have inaccurate knowledge of) how frequently their children experience online risks (Byrne et al., 2014; Caivano et al., 2020; Dehue et al., 2008; Symons et al., 2017), including parents in Ireland (McGuire & O’Higgins Norman, 2017; O’Neill et al., 2011). However, which parental factors contribute to this lack of awareness have yet to be identified (Caivano et al., 2020).

### Parental Self-Efficacy in Securing Children’s Online Safety

In descriptive research on how parents approach cyberbullying and online safety in Ireland, about 75% (*N* = 908) reported high confidence in their awareness (McGuire & O’Higgins Norman, 2017). This self-perceived confidence has been consistently reported in a series of descriptive studies over the last decade. In the National Advisory Council Survey for Online Safety (NACOS, 2021) as well as the EU Kids Online survey (O’Neill et al., 2011), most of the participating parents from Ireland reported high confidence in their own ability as well as the child’s ability to cope with online risks Among 25 European countries, the parent sample from Ireland (*N* = 1000) reported the second highest level of confidence in their awareness (Livingstone et al., 2011). Most participating Irish parents (74%) were confident that “it is not very or at all likely that their child will encounter anything that bothers them in the next six months” (O’Neill et al., 2011, p. 49). However, research over the last decade (NACOS, 2021; O’Neill et al., 2011) revealed that only one in three parents among the sample from Ireland were aware of their children’s exposure to online risks related to pornography, grooming, or self-harm. For instance, 37 out of 40 (O’Neill et al., 2011) or 65 out of 92 parents among the sample from Ireland (NACOS, 2021) were unaware their child met face-to-face somebody they first got to know online. Compared to parents across 24 European countries, most of the participating parents from Ireland (68%) were least likely to recognize their children had an online risk experience; they appeared to be the least aware of their children’s online risks (Livingstone et al., 2011). These findings seem to indicate that parental self-efficacy in securing their children’s online safety is a determinant of parental awareness (or lack thereof) of online risks. As such, what can parents do for their children’s Internet use in order to be aware of and prevent various online risks?

### Parental Mediation of Children’s Internet Use

Research on parental mediation of children’s Internet use is a continuation of studies on television viewing and video gaming, especially the study by Valkenburg et al. (1999), who proposed three categories: (a) active mediation (i.e., verbally explaining and encouraging proper use), (b) restrictive mediation (i.e., setting rules for children's television viewing behavior), and (c) co-viewing mediation (i.e., observing the child’s behavior while watching television together either next to the child or in the same room). However, it is unclear whether this trichotomy of parental mediation is also applicable to children’s Internet use.

There is no conformity to the distinction in parental mediation strategies (Caivano et al., 2020; Livingstone & Helsper, 2008; Nikken & Jansz, 2014; Sonck et al., 2013), but common to all the categorizations is *active versus restrictive* mediation (Symons et al., 2017). Livingstone et al. (2017) tested the factor structure of five parental mediation strategies and proposed a two-factor model of enabling and restrictive mediation, which has yet to be confirmed in terms of construct validity across countries (Kuldas et al., 2021). A recent review of 10 parental mediation scales (Kuldas et al., 2021) found no consistent evidence for the content and construct validity of a dichotomy, trichotomy, or any other model of parental mediation strategies. For instance, in contrast to the proposed model for the UK context, which considered parental monitoring and technical control as enabling mediation, research in the USA context operationalized these constructs as restrictive mediation (Kuldas et al., 2021). Therefore, Kuldas and colleagues argued that the lack of conformity is attributable to the inaccurate conceptualization of parental mediation (Kuldas et al., 2021).

### Conceptualization

It is unclear whether parental awareness is an outcome or antecedent of parental mediation. A review of this issue shows that most earlier studies conceptualized parental awareness as an antecedent rather than the outcome of parental mediation (Racz & McMahon, 2011). Recent research (Caivano et al., 2020) acknowledged that it remains unclear if parents set rules as a result of being aware or with the hope of becoming aware of their children’s online risk experiences. However, this question does not suggest the conceptualization of parental awareness as an antecedent, mainly because setting rules with the hope of *becoming aware* is not parental awareness but mediation (Kuldas et al., 2023).

Therefore, the present research argues that parental awareness of children’s online risks is an outcome of parental mediation strategies, either in the *past* (being aware) or in the *future* (becoming aware). What parents do to become aware of their children’s online risks suggests parental meditation is an antecedent of their awareness. What strategy parents choose after they are aware of online risks makes parental mediation an outcome. Accordingly, parental mediation comes first, then parental awareness, and, in turn, the same or another mediation strategy is used. The research hereby acknowledges a conceptualization of bi-directional relationships between parental awareness and mediation strategies (Kuldas et al., 2023).

However, not only the bi-directional conceptualization but also the unidirectional one has yet to be tested. A series of studies over the last decade (Caivano et al., 2020; Criss et al., 2015; Lippold et al., 2014; Symons et al., 2017) conceptualized parental awareness as an outcome of parental mediation, but without testing it. This conceptualization is in line with Dishion and McMahon’s (1998) approach, which postulates that parental monitoring of *offline* behaviors differentiates the degree of parental awareness. However, the approach has yet to be tested for children’s *online* behavior. Although Symons et al. (2017) provided limited evidence for the approach to the online context, they did not test the conceptualization. Caivano et al. (2020) were also not able to determine whether restrictive mediation was a result or antecedent of parental awareness. The present study has adapted Dishion and McMahon’s (1998) approach to test the unidirectional conceptualization.

### Conceptual Framework

**Fig. 1:**
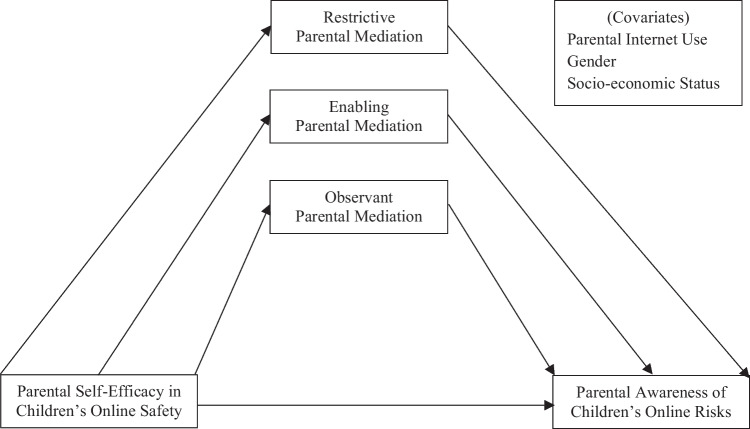
A conceptual framework for parental awareness of children’s online risks

The current research is delimited to one direction towards parental awareness. Figure 1 depicts this conceptual framework, where to have *Confidence*, to *Do*, and to *Know* are respectively an antecedent, a defining attribute, and a consequence of parental awareness of children’s online risks. The research is not focused on how parental awareness in turn determines bi-directional effects between parental self-efficacy and mediation strategies. Parental self-efficacy can determine and be determined by parental mediation strategies (Glatz et al., 2018). Parental mediation and awareness may result in increased self-efficacy in parenting for children’s online safety (Glatz et al., 2018).

#### From Parental Self-Efficacy to Awareness

Although parents are more aware of their children’s experiences as a victim of cyberbullying than other online risks, they tend to underestimate how frequently it happens (Caivano et al., 2020; Symons et al., 2017). Recent evidence falls short of explaining why parents tend to underestimate it. However, there is a growing argument that it happens due to parents’ self-efficacy (Australian Office of the eSafety Commissioner, 2018; Glatz et al., 2018; Livingstone et al., 2011; O’Neill et al., 2011) and the ability for Internet use (Vandoninck et al., 2013). Therefore, parents’ self-efficacy is likely to determine their underestimation or overestimation of the frequency of their children’s exposure to online risks (Hypothesis 1).

#### From Parental Self-Efficacy to Mediation

Parental self-efficacy directly affects (McGuire & O’Higgins Norman, 2017) and is affected by their mediation strategies for their children’s online safety/risks (Glatz et al., 2018). Although higher levels of self-efficacy are associated with all the parental mediation strategies (Glatz et al., 2018), further evidence indicates that parents with high self-efficacy are less likely to choose restrictive mediation (McGuire & O’Higgins Norman, 2017). The present study aims to further explore the relationship between parental self-efficacy and mediation strategies. The self-efficacy of parents is likely to predict their restrictive (Hypothesis 2), enabling (Hypothesis 3), and observant parental mediation (Hypothesis 4).

#### From Parental Mediation to Awareness

After setting rules (restricting), supervising (enabling), or co-using (observing), parents may become aware of online risks (Symons et al., 2017). However, only a few studies (Byrne et al., 2014; Cerna et al., 2016; Australian Office of the eSafety Commissioner, 2018) suggested enabling mediation, in the form of parent–child communication on online safety, as the best predictor of parental awareness. Children’s perception of enabling but not restrictive mediation was linked to an increased probability of the disclosure about their experience as a victim of cyberbullying behavior (Cerna et al., 2016). In an earlier study (Byrne et al., 2014), this probability decreased when the child perceived difficulty in the child-parent communication. Therefore, Symons et al. (2017) argued that because active/enabling mediation involves open parent–child communication, it could be the best strategy for parental awareness, but their own study showed no supportive evidence. In contrast, they found limited evidence for the relationship between parents’ restrictive mediation and accurate awareness. However, empirical evidence for restrictive mediation is also inconclusive. For example, Caivano et al. (2020) found no relationship between restrictive mediation and parental awareness. Hence, further research is needed to test whether restrictive (Hypothesis 5), enabling (Hypothesis 6), and observant mediation (Hypothesis 7) predict parental awareness of their children’s online risks.

#### From Parental Self-Efficacy to Awareness Through Mediation

Some evidence indicates that parents who have high self-efficacy are less likely to choose restrictive mediation (Duerager & Livingstone, 2012), which might be an underlying reason for their lack of awareness. Therefore, McGuire and O’Higgins Norman (2017) argued that if Irish parents with high self-efficacy engaged in restrictive instead of active and observant mediation, they would be aware of how frequently their adolescent engaged in cyberbullying. However, other studies showed that most parents who always or usually set rules for their children’s Internet use underestimated how frequently their adolescent was a victim or perpetrator of cyberbullying (Dehue et al., 2008). Moreover, such parents might have lower self-efficacy (Coleman & Karraker, 2000). Accordingly, the association of either low or high self-efficacy with parental awareness has yet to be accurately estimated, testing whether this association is mediated by restrictive (Hypothesis 8), enabling, (Hypothesis 9), and observant (Hypothesis 10) mediation.

#### Covariates

Parents’ mediation strategies vary according to their socioeconomic status, gender, and the ability for using the Internet (Kuldas et al., 2021). Therefore, these parental characteristics were included in the conceptual model as covariates. For instance, parents who are familiar with digital technology are likely to have more confidence in mediating their children’s online activities. Such parental confidence was reported by the majority of a convenience sample of parents (80% of *N* = 908) in Ireland (McGuire & O’Higgins Norman, 2017).

## Methods

### Procedures and Ethical Considerations

In Autumn 2019, all the post-primary schools in Ireland (*N* = 730) were requested to invite the parents of the school students to fill out an online survey on parental awareness of their child’s online risks (e.g., being a target or engaged in cyberbullying behavior). However, only twenty-eight school principals agreed to partake in the study and, thus, received a private email containing a direct link to the plain language statement, consent form, and the survey itself, so as to forward it to the parents. Each parent was informed about ethical principles (e.g., anonymity, confidentiality, and the right to withdraw from the survey at any time) and signed a consent form. Ethical principles were approved by the authors’ university’s ethics committee.

As this research was conducted anonymously with adults, the ethical issues involved were somewhat minimal compared to other studies that involved children. However, the research was conducted online, and this did raise some ethical issues. One ethical issue that has been identified by other researchers is the issue of participant age verification (Hokke et al., 2018). When research is conducted anonymously online, there is a greater need to either verify or trust the age of the participants. In the case of this study, participants were those who were recruited through post-primary schools as the parents of second-year students (14 years of age) and, as such, were extremely likely to be adults over 30 years of age. The other ethical issue that warranted consideration was the use of the Internet as a means of data collection. In terms of privacy, confidentiality, and anonymity, some researchers have raised concerns about using social media sites as a mechanism to recruit participants. Apart from any potential risks linked to how social media sites may process participant data, other risks include participants compromising participant recruitment through sharing about their participation online, possibly resulting in some participant bias, and vulnerability (Hokke et al., 2018; Parsons, 2015). Therefore, it was decided that participants for this study would not be recruited through social media sites, but only through direct contact emails held by their child’s school.

### Participants

A convenience sample of 388 parents of 14-year-old adolescents in post-primary schools in Ireland participated in the survey (53 Fathers, 335 Mothers). The socio-economic status (SES) of parents was estimated at three levels based on their self-reported educational level and work status: low SES (*n* = 70), medium SES (*n* = 106), and high SES (*n* = 212).

### Measures

#### Parental Mediation of Children’s Internet Use

A 14-item scale measuring parental mediation strategies was adapted from the Australian Office of the eSafety Commissioner (2018). Parents ranked their *agreement* on a six-point scale, ranging from 1 (Strongly disagree) to 6 (Strongly agree), about what strategies they follow for their child’s Internet use.

#### Parental Awareness of Children’s Online Risks

A 15-item scale was adapted from the Australian Office of the eSafety Commissioner (2018). Parents were asked about the extent to which they were aware of how frequently their children had faced risks online in the last school term. Parents ranked their awareness of the *frequency* on a five-point scale: “It hasn’t happened in the past couple of months (1),” “It has happened only once or twice (2),” “2 or 3 times a month (3),” “About once a week (4),” and “Several times a week (5).”

#### Parental Self-Efficacy in Children’s Online Safety

An 8-item scale for the internet-specific parental self-efficacy was adopted from Glatz et al. (2018). The scale started with the question “How confident do you feel in your ability to prevent your child from…,” followed by eight items describing various online risks. Parents rated their own *confidence* on a five-point scale from 1 (Not confident) to 5 (Extremely confident).

#### Parental Internet Use

A total of 22 items in two sets were used to measure parental Internet use. The first set included five items, adapted from the Australian Office of the eSafety Commissioner (2018), to measure the frequency of using different devices for the Internet. The second set included the rest of the items that measured how frequently parents use different social media applications. All the items were rated on a five-point scale from 1 (Not at all) to 5 (Daily).

### Statistical Analyses

#### Minimum-Rank Factor Analysis (MRFA)

The factorial structure, construct validity, and composite reliability of the *parental mediation scale* were estimated in three steps. The factorial structure was tested via a MRFA of the polychoric correlation matrices, using the FACTOR (Version 10.10.03) statistical program (Ferrando & Lorenzo-Seva, 2017). To retain an optimal number of factors, the *Hull method* with the adjustment index *Common Part Accounted for* was used, as it is one of the best methods when there is a violation of normality (Ferrando & Lorenzo-Seva, 2017). Next, convergent validity as the Average Variance Extracted (AVE > 0.50) and discriminant validity as the square root of AVE greater than all the inter-factor correlations (Fornell & Larcker, 1981) were estimated to satisfy two criteria for construct validity (Hair et al., 2014). Last, composite reliability was estimated as a measure of internal consistency (Hair et al., 2014). These three steps can be considered to be testing a reflective measurement model, and testing relationships between a latent construct and its reflective indicators (Lewis et al., 2005).

#### Two-Parameter-Logistic (2PL) Model of Item Response Theory (IRT)

The 2PL model of IRT, using the *ltm* package in R (Rizopoulos, 2006), was used for the estimation of item difficulty and discrimination parameters, thereby testing the construct validity and reliability of the three scales: (a) parental self-efficacy in children’s online safety (6-item), (b) parental awareness of children’s online risks (14-item), and (c) parental internet use (8-item). *Test characteristic curve* (TCC) was used to determine whether individuals’ latent characteristics were based on their true scores on each scale (Baker, 2001). TCC allowed to estimate the cutoff points for low and high levels of parental awareness, self-efficacy, and Internet use. Before these analyses, four assumptions for an IRT analysis (unidimensionality, local independence, monotonicity, and measurement invariance) were met.

#### Logistic Regression Analysis of a Multiple-Parallel Mediation Model

PROCESS macro (Hayes, 2018), an extension of IBM SPSS v.25 statistical software, was used to test the mediation model because it allows for a logistic regression analysis of a dichotomous dependent variable (Hayes, 2018). The initial model consisted of one independent variable (parental self-efficacy), three mediating variables (enabling, restrictive, and observant parental mediation), and one dichotomous dependent variable (parental awareness). There were also three covariates (parents’ Internet use, gender, and SES). Indirect effects of parental self-efficacy on parental awareness were tested with 95% bias-corrected bootstrap confidence intervals (non-parametric bootstrapping), derived from 5000 resamples (Hayes, 2018).

### Coding

To test the IRT model, responses on the five-point scores were dichotomized based on two criteria: (a) whether the dichotomization fits the item difficulty and discrimination parameters, and (b) the dichotomized item scores on each scale were summed up to discriminate between lower and higher levels of the latent constructs. An initial item parameter analysis indicated a cutoff point for distinguishing between parental (a) unawareness (0, it hasn’t happened in the past couple of months) and awareness (1, it has happened only once or twice–or more); (b) low self-efficacy (0, not confident–a little confident–somewhat confident) and high self-efficacy (1, very confident–extremely confident); (c) low Internet use (0, not at all–once per week–a few times per week) and high Internet use (1, several times per week–daily). These dichotomized scores on each scale were separately summed up for the TCCs to determine levels of parental awareness, self-efficacy, and Internet use.

## Results

### The Parental Mediation Scale: Factorial Structure, Construct Validity, and Composite Reliability

The MRFA of the parental mediation scale suggested the exclusion of three items from the 14-item scale due to low loadings (< 0.30). The Kaiser–Meyer–Olkin measure verified the sampling adequacy of the remaining 11 items (ΚΜΟ = 0.80, the Bartlet test, *p* < 0.001). The Hull method (adopting the 1% rule accounting for the common part of the index) advised a three-common-factor solution with the highest value of goodness of fit (*f*(25) = 0.453) and scree test (4.306, *p* < 0.000). The three factors explained 85.63% of the common variance. Factor 1, 2, and 3 accounted for 53%, 22%, and 11% of the explained variance, respectively. Composite reliability (CR > 0.70), convergent validity (AVE > 0.50), and discriminant validity (the square root of AVE > the inter-factor correlations, Fornell & Larcker, 1981) satisfied the criteria for internal consistency and construct validity (Hair et al., 2014). Table 1 displays the rotated factor loadings of 11 items of the parental mediation scale as well as the estimated values for its composite reliability, convergent validity, and discriminant validity, and the highest inter-factor correlation values.

**Table 1:** Results from an exploratory factor analysis of the parental mediation scale

Item	Factor loading		
	1	2	3
**Factor 1: enabling parental mediation**			
I speak to my child about being respectful to others online	.96		
I talk to my child regularly about online risks and what to do	.86		
I listen to my child’s online social problems, if they have any	.72		
**Factor 2: observant parental mediation**			
I can see what is on their screen		.86	
They are where I can see them		.81	
I check on them intermittently while they are using the device		.81	
**Factor 3: Restrictive parental mediation**			
I have direct access to all of my child’s personal online accounts			.68
I use age guidelines in relation to my child’s use of social media, apps and games			.66
I monitor their internet use (check browsing history, view their social media accounts)			.75
We have enough controls in place (filters, internet settings, etc.) so that I don’t need to supervise directly			.77
I limit the amount of time my child spends online (e.g., disable data access, remove devices from bedrooms etc.)			.67
Composite reliability	.89	.87	.83
Convergent validity	.73	.68	.50
Discriminant validity	.85	.82	.71
Inter-factor correlations (maximum value)	.41	.59	.59

*Ν* = *336.* The extraction method was the Minimum-Rank Factor Analysis. Items with factor loading below .30 were excluded. Items were adapted from the Australian Office of the eSafety Commissioner (2018). Convergent validity was estimated via Average Variance Extracted (AVE = the sum of the squared loadings divided by the number of indicators). Discriminant validity was estimated via the square root of AVE greater than inter-factor correlations

First, the three items that only loaded on Factor 1 with high composite reliability (0.89) reflect *Enabling Parental Mediation*, defined as parental consideration of their child as *agentic online* (i.e., addressing the child’s sense of agency over both opportunities and risks online) by regularly encouraging and instructing the child to disclose any risk experience or to recognize an online risk and what to do against it (Kuldas et al., 2021). Next, the three items that only loaded on Factor 2 with composite reliability (0.87) reflect *Observant Parental Mediation*, defined as when a parent intermittently observes—is intermittently alert, watchful, or attentive to—both the child’s behavior and the screen (e.g., smartphone, tablet, or computer) when online (Kuldas et al., 2021). Last, the five items that only loaded on Factor 3 with high composite reliability (0.81) reflect *Restrictive Parental Mediation*, which refers to setting rules (for the child’s Internet use of social media, apps, or games), filters (technical restriction to online contents), and limits (the Internet access), while having direct access (monitoring or checking) to the child’s personal accounts and browsed history (Kuldas et al., 2021).

### The Parental Awareness, Self-Efficacy, and Internet Use Scales: Construct Validity and Reliability

**Fig. 2:**
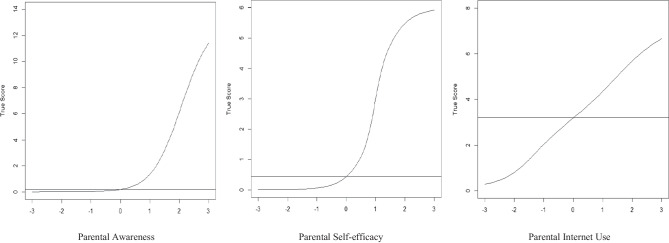
Test characteristic curves of true scores on the parental awareness, self-efficacy, and internet use scales

The construct validity of the three scales has been established via the IRT-2PL model, indicating the discrimination ability of each item (see Table 2) and each scale (see Fig. 2). Following this result, the criterion for reliability (internal consistency) was satisfactorily met, based on McDonald’s (1999) categorical Omega (ω) values of the scale for the parental awareness (categorical-ω = 0.94), self-efficacy (categorical-ω = 0.94), and Internet use (categorical-ω = 0.78), using Robust Diagonally Weighted Least Squares (RDWLS) estimations of the polychoric correlation matrices as implemented in the FACTOR program (Ferrando & Lorenzo-Seva, 2017).

**Table 2:** Results from IRT 2-PL model analyses of the parental awareness, self-efficacy, and internet use scales

Item	$\alpha$	*z*	DIF Lord’s *χ*^2^	Categorical-ω
**Parental awareness scale**				.94
Had lies or rumors spread about them	1.75	4.82	0.72	
Had inappropriate personal information posted without their consent	2.10	3.87	0.68	
Had inappropriate photos of them posted without their consent	1.92	3.50	0.21	
Been called insulting names	2.17	5.28	2.82	
Been socially excluded	2.02	5.31	2.87	
Received threats	2.30	4.43	1.24	
Had someone pretend to be them online	1.52	3.29	0.25	
Had their accounts accessed without their permission	2.00	4.25	0.09	
Received repeated unwanted communications	1.94	5.10	3.13	
Been contacted by strangers	1.50	4.36	3.42	
Their personal information was used in a way they did not like	2.03	4.05	1.11	
Had something distressing about them disclosed to others	2.34	4.06	1.76	
Had pornography shown/sent to them	1.58	4.18	3.94	
Had or violent or racist content shown/sent to them	1.82	4.42	2.78	
**Parental self-efficacy scale**				.94
…coming in contact with dangerous persons	1.61	7.17	4.90	
…being bullied	1.36	6.36	2.47	
…coming in contact with material that will make him/her upset	1.50	5.74	1.09	
…ending up on a website with pornographic content	2.14	7.18	0.30	
…ending up on a website that has hatred content against individuals or groups	2.13	7.23	2.80	
…giving out or posting personal information online that could be problematic for safety reasons	1.65	7.12	4.07	
**Parent’s internet use scale**				.78
Desktop or laptop computer	0.73	4.25	2.99	
Smartphone	2.51	3.93	2.91	
Smart TV (including services like YouTube)	0.76	3.86	2.36	
Facebook	0.96	4.75	3.10	
Instagram	1.72	4.15	2.69	
Twitter	1.53	3.81	1.12	
Snapchat	0.94	2.38	1.17	
WhatsApp	1.50	4.97	0.79	

Retained only items with a discrimination parameter $(\alpha\geq0.5)$. Non-significant *DIF* Differential Item Functioning (*p* < 0.001) for the SES levels, ω McDonald’s categorical Omega

First, for the *Parental Awareness Scale*, the discrimination parameters for all 14 items were significant, allowing these items to be retained (*α* > 0.05, *z* > 1.96). Its TCC identified a cutoff point of 0.2 as the true score, which indicates that reporting *one risk* incident is a sufficient indication of the parental awareness. Scores above this cutoff point indicate higher awareness.

Second, for the *Parental Self-Efficacy Scale*, the discrimination parameters for six out of eight items were significant, and therefore, these items could be retained (*α* > 0.05, *z* > 1.96). Its TCC showed a cutoff point of 0.45 as the true score, indicating that parental self-efficacy in tackling *one risk* incident is sufficient to distinguish between low (0, none of the risks), moderate (1, one to three risks), high (2, four to five risks), and higher (3, six risks) self-efficacy.

Third, for the *Parental Internet Use Scale*, the discrimination parameters for eight out of 22 items were significant, and therefore, these items could be retained (*α* > 0.05, *z* > 1.96). Its TCC suggested a cutoff point of 3.2 as the true score, indicating low parental Internet use among parents who endorsed a maximum of three items. Higher Internet use was among parents who endorsed four or more items.

### Logistic Regression Analysis of a Multiple Parallel Mediation Model

**Fig. 3:**
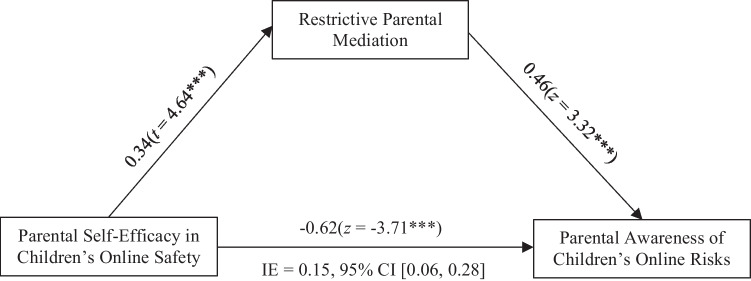
A logistic regression analysis of the mediation model of parental awareness of children’s online risks

To facilitate the interpretation of the results, the direct effects are reported first (see Table 3) and then the indirect effects (see Fig. 3).

#### Direct Effect of Parental Self-Efficacy on Awareness

There was a collective significant effect of parental self-efficacy on their awareness of the frequency of the child’s online risk experiences, after controlling for their enabling, restrictive, and observant mediation as well as Internet use, gender, and SES. The direct effect of parental self-efficacy on their awareness was negatively significant (*b* = − 0.62, *z* = − 3.71, *p* < 0.001), indicating that parents scoring higher on the self-efficacy reported no incident of the child’s online risks. Those scoring lower on self-efficacy reported one, two, or more incidents.

#### Direct Effect of Parental Self-Efficacy on Enabling Mediation

There was a collective significant effect of parental self-efficacy on their enabling mediation, while controlling for their Internet use, gender, and SES, *F*(4, 340) = 3.8, *p* = 0.005, *R*^*2*^ = 0.04. The direct effect of parental self-efficacy on their enabling mediation was positively significant (*b* = 0.14, *t* = 2.76, *p* = 0.006), indicating that parents scoring higher on self-efficacy were more likely to practice enabling mediation than those scoring lower. Among the covariates, their Internet use (*b* = 0.08, *t* = 0.94, *p* = 0.35) and SES (*b* = − 0.02, *t* = − 0.35, *p* = 0.73) were not significantly related to enabling mediation, but gender was. Mothers were 1.38 times more likely to choose the enabling mediation strategy than fathers (*b* = 0.32, *t* = 2.73, *p* = 0.007).

#### Direct Effects of Parental Self-Efficacy on Restrictive Mediation

There was a collective significant effect of parental self-efficacy on restrictive mediation, while controlling for parents’ Internet use, gender, and SES: *F*(4, 340) = 7.51, *p* < 0.001, *R*^*2*^ = 0.08. The direct effect of parental self-efficacy on restrictive mediation was positively significant (*b* = 0.34, *t* = 4.64, *p* < 0.001), indicating that parents scoring higher on self-efficacy were more likely to practice restrictive mediation than those scoring lower. Among the covariates, parental Internet use (*b* = 0.15, *t* = 1.31, *p* = 0.19) and SES (*b* = − 0.03, *t* = − 0.41, *p* = 0.68) were not significantly related to restrictive mediation, but gender was. Mothers were 1.62 times more likely than fathers to choose the restrictive mediation strategy (*b* = 0.48, *t* = 2.87, *p* = 0.004).

#### Direct Effects of Parental Self-Efficacy on Observant Mediation

The effect of parental self-efficacy on observant mediation was not significant, while controlling for their Internet use, gender, and SES: *F*(4, 340) = 2.29, *p* = 0.06.

#### Direct Effects of Enabling—Restrictive—Observant Mediation on Awareness

The direct effects of enabling (*b* = 0.21, *z* = 1.18, *p* = 0.24) and observant mediation (*b* = − 0.11, *z* = − 1.18, *p* = 0.24) on parental awareness were not statistically significant. Only restrictive mediation had a direct positive effect (*b* = 0.46, *z* = 3.32, *p* < 0.001); parents scoring higher on restrictive mediation were more likely to be aware of how frequently their child encountered online risks. Among the covariates, there was no statistically significant relationship with parental awareness (see Table 3).

**Table 3:** Results for the direct effects in the multiple-parallel mediation model of parental awareness of children’s online risks

								95% B-C CI	
Hypotheses					*SE*	*t*	*z*	*LL*	*UL*
Parental self-efficacy	→	Enabling mediation	0.14	0.05		2.76**		0.04	0.24
Gender	→		0.32	0.12		2.73**		0.09	0.55
Socio-economic status	→		−0.02	0.05		−0.35		−0.12	0.08
Parental internet use	→		0.08	0.08		0.94		−0.08	0.23
Parental self-efficacy	→	Restrictive mediation	0.34	0.07		4.64***		0.19	0.48
Gender	→		0.48	0.17		2.87**		0.15	0.81
Socio-economic status	→		−0.03	0.07		-0.41		−0.18	0.12
Parental internet use	→		0.15	0.11		1.31		−0.07	0.38
Parental self-efficacy	→	Observant mediation	0.20	0.10		1.98		0.00	0.41
Gender	→		0.45	0.24		1.87		−0.02	0.92
Socio-economic status	→		0.18	0.11		1.74		−0.02	0.39
Parental internet use	→		−0.01	0.16		-0.01		−0.32	0.32
Parental self-efficacy	→	Parental awareness	−0.62	0.17			−3.71***	−0.95	−0.29
Enabling mediation	→		0.21	0.18			1.18	−0.14	0.57
Restrictive mediation	→		0.46	0.14			3.32***	0.19	0.73
Observant mediation	→		−0.11	0.09			−1.18	−0.29	0.07
Gender	→		0.40	0.39			1.03	−0.36	1.15
Socio-economic status	→		0.03	0.16			0.19	−0.28	0.33
Parental internet use	→		−0.04	0.24			−0.15	−0.50	0.43

*b =* Unstandardized Estimates, *B-I-CI* Bias-Corrected Confidence Interval, *LL =* lower limit, *UL =* upper limit

^**^p < .01; ***p < .001

#### Indirect Effect of Parental Self-Efficacy on Awareness

The logistic regression analysis of the multiple-parallel mediation model showed a significant and positive indirect effect of parental self-efficacy on their awareness through restrictive mediation, IE = 0.15, 95% CI [0.06, 0.28]. The model-fit statistics with values of F/-2 Log-Likelihood (model χ2 improvement) = 420.83 (28.63, *p* < 0.001), Nagelkerke *R*^*2*^ = 0.11, and Cox and Snell *R*^*2*^ = 0.08 indicated a good model fit (see Fig. 3). Because of their non-significant direct effects on parental awareness, parents’ Internet use, SES, gender (covariate variables), enabling mediation, and observant mediation were not included in the final mediation analysis.

As the result shown in Fig. 3 indicates, parents scoring higher on self-efficacy were more likely to be aware of how frequently their child faced online risks when they engaged in more restrictive mediation. This positive indirect effect was in contrast to the negative direct effect of high self-efficacy on parental awareness.

## Discussion

Children’s use of the Internet, especially social media, comes with both opportunities and risks, which have created substantial changes in parental mediation strategies and awareness. A lack of parental awareness is an obstacle that prevents parents from accommodating their mediation strategies to their children’s needs for online safety (Caivano et al., 2020). To understand such challenges warrants empirical research on what parents can do for their children’s purposeful use of the Internet, while preventing online risks (Nikken & Jansz, 2014; Symons et al., 2017). The scarcity of empirical research on parental mediation falls short of suggesting what parents can do to become aware of online risks and keep their children safe when using the Internet (Caivano et al., 2020; Symons et al., 2017). An accurate measurement and a conceptual framework of the parental factors that increase or decrease parental awareness were required (Symons et al., 2017).

The novelty of the present research has manifested itself in closing the contextual, empirical, and methodological gaps by the proposed conceptual framework of parental awareness after testing the construct validity and reliability of the scale for (a) parental self-efficacy, (b) parental awareness, (c) parental Internet use, and (d) parental mediation. The construct validation, based on the 2PL model of IRT, has yielded a cutoff point for distinguishing between lower and higher levels of parental awareness and self-efficacy. Parental self-report of one risk incident is a sufficient indication of their awareness of children’s online risk experiences. Parental confidence in preventing even one risk incident is a sufficient indication of their self-efficacy.

### The Higher Self-Efficacy, the Less Awareness

Although earlier studies found most parents were unaware of how frequently their children experienced online risks (Dehue et al., 2008; McGuire & O’Higgins Norman, 2017; Symons et al., 2017), they lacked statistical evidence for its association with parental self-efficacy. One reason is likely to be the scarcity of measurement scales with adequate psychometric properties. In the current study, the construct validity and reliability of the parental self-efficacy and awareness scales have been established. Another reason could be the lack of a conceptual framework for parental awareness, due to which recent studies (Caivano et al., 2020; Symons et al., 2017) fell short of explaining: Why do parents underestimate how frequently their children experience risks online? The present research has tested a conceptual model and found that the underestimation is related to parental self-efficacy. The higher parental self-efficacy in children’s online safety, the lower parental awareness of children’s online risks among the sample of Irish parents.

The present inferential statistical evidence substantiates descriptive findings by the National Advisory Council Survey for Online Safety (NACOS, 2021), Anti-Bullying Centre in Dublin (McGuire & O’Higgins Norman, 2017), and EU Kids Online survey (O’Neill et al., 2011). In these studies, most parents surveyed in Ireland appeared to have high self-efficacy but were unaware of their children’s online risk experiences. For example, the parent sample reported the second highest level of self-efficacy but appeared to be the least aware that their child met face-to-face a stranger they first met online (O’Neill et al., 2011) as compared to 24 other European countries (Livingstone et al., 2011). Hence, parents’ higher confidence in their ability to prevent their children from facing online risks might be a risk factor inhibiting parental awareness.

### The Higher Self-Efficacy, the More Restrictive and Enabling Mediation

Higher parental self-efficacy appeared to lead to more restrictive and enabling mediation. In contrast to earlier research (McGuire & O’Higgins Norman, 2017), the current study found that parents with lower levels of self-efficacy were engaged in less restrictive and enabling mediation. This finding is inconsistent with the earlier descriptive research, in which two-thirds of Irish parents with higher self-efficacy reported no restrictive mediation, but only a form of enabling mediation (McGuire & O’Higgins Norman, 2017). This is attributable to their lack of self-efficacy in restrictive mediation, given that the majority of Irish participants reported not knowing how to use restrictive prevention tools for their children’s Internet use (McGuire & O’Higgins Norman, 2017). As such, enabling mediation is likely to be a more common practice than restrictive mediation among parents with higher self-efficacy in Ireland. However, parents with higher self-efficacy might engage in both enabling and restrictive mediation to elicit more information about their children’s online behaviors (Glatz et al., 2018), although the present research found no positive association between higher self-efficacy and restrictive, enabling, or observant mediation.

### The More Restrictive Mediation, the More Parental Awareness

Whether parental awareness is an outcome or antecedent of mediation strategies has been a recurring issue (Symons et al., 2017). Earlier studies conceptualized parental awareness as an antecedent (Racz & McMahon, 2011). In contrast, recent studies (Criss et al., 2015; Lippold et al., 2014; Symons et al., 2017) defined parental awareness as an outcome. Supporting the recent conceptualization, the present mediation analysis revealed that parental awareness is an outcome of restrictive mediation.

Which parental mediation strategy best predicts their awareness is unclear in recent studies (Caivano et al., 2020; Symons et al., 2017), which are very few. Unlike earlier studies (Byrne et al., 2014; Cerna et al., 2016; Australian Office of the eSafety Commissioner, 2018) suggesting enabling mediation as the best practice, the present research found that only restrictive mediation is likely to predict parental awareness. Similar to recent research (Symons et al., 2017), there was no evidence for an association of enabling and observant mediation with parental awareness. In the recent research in Belgium (Symons et al., 2017), none of the parental mediation strategies (i.e., active tracking, supervision/co-use, interaction restriction, and access restriction) including open parent–child communication could be associated with parental awareness. However, there was limited evidence for an association between parents’ accurate awareness and restrictive mediation (Symons et al., 2017). This and the current finding for the association of restrictive mediation with parental awareness are inconsistent with very recent studies, which found no evidence for this association (Caivano et al., 2020).

### The Higher Self-Efficacy, the More Restrictive Mediation and Awareness

When parents have higher self-efficacy, restrictive mediation appears to be the best strategy for their awareness. In the present research, the restrictive, but not enabling and observant, mediation positively mediated the relationship between parental self-efficacy and awareness. As a function of their restrictive mediation, parents who had higher self-efficacy reported higher awareness, while those with lower self-efficacy reported lower awareness. This empirical finding substantiates McGuire and O’Higgins Norman’s (2017) argument; if confident parents in Ireland engage in restrictive mediation, they would be more aware of the frequency of their children’s online risk experiences. The indirect finding for this argument opposes the earlier finding of the negative direct effect of parental self-efficacy on their awareness. Hence, parents who had higher self-efficacy in their children’s online safety were more likely to underestimate how frequently their adolescent was a victim of online risks. In contrast, parents with lower self-efficacy were more likely to overestimate it. These findings are inconsistent with recent studies, which found no difference between parents using and not using restrictive mediation (Caivano et al., 2020; Symons et al., 2017). In these studies, although both restrictive and non-restrictive parents were relatively aware of their children’s experiences as a victim of cyberbullying, both groups underestimated how frequently it happened.

Such inaccurate estimations or self-perceptions of competence are attributable to the Dunning–Kruger effect (Dunning, 2011; Kruger & Dunning, 1999), such as when parents have high confidence in their ability to prevent online risks or secure online safety for their children but lack awareness of how frequently their children have experienced an online risk. This effect might ensue from their perceptions of parental mediation strategies. When enabling mediation is preferred or perceived as easy (i.e., demanding less cognitive effort or low digital literacy skills), parents are likely to rate their self-efficacy and awareness high, thereby disclosing the Dunning–Kruger effect (i.e., the more enabling mediation, the higher overestimation of parental awareness). In contrast, when restrictive mediation is perceived as difficult, parents with low self-efficacy are likely to rate their awareness as low.

## Limitations

An essential weakness of the mediation analysis of a cross-sectional survey is that causality cannot be inferred (Hayes, 2018). Therefore, the nature of a cross-sectional survey does not afford causal inferences from the findings. However, this weakness is not peculiar to the current research. In general, researchers do not use statistical methods (e.g., linear regression models) to make causal inferences about findings from cross-sectional research designs (Hayes, 2018). Instead, the findings can be interpreted as preliminary evidence for the likely co-variation of the variables in the model, which a longitudinal or experimental study can test for a causal inference.

Further limitations that restrict the generalizability are mostly due to five reasons related to the convenience sampling method, sample size, and lack of evidence for the effects of parent-risk characteristics, which include parental perception of seriousness, quantity, and type of online risks as well as age, gender, SES, country of residence (nationality/race/ethnicity), childrearing culture/values, and digital literacy skills of parents. First, convenience sampling could exclude parents who lacked digital literacy skills or did not (want to) use the Internet to participate in an online survey. Second, the parent-risk characteristics could determine what mediation strategy parents choose and how confident they feel *before* or *after* awareness. For example, parents may need different digital knowledge and skills to prevent a specific online risk like piracy than other types of risks, such as cyberbullying, cybervictimization, or unwanted sexting. As every online risk is inherently different, parents need different knowledge and skills. Another example, parental awareness is likely to vary according to the maternal and paternal roles in children’s online safety. Mothers and fathers in Ireland might differ in their perception of the seriousness and type of online risks, thereby affecting their self-efficacy, mediation strategy, and awareness (McGuire & O’Higgins Norman, 2017). However, no conclusion for such a cross-gender comparison is deducible from the findings, due to the lack of measurement invariance in the self-reports of participating mothers and fathers.

Third, given that almost one-tenth of parents were fathers, this sample size might account for the lack of measurement variance, which is highly likely to be an issue in previous studies, as well. There was no report of measurement invariance in similar research in Ireland, in which mothers made up 88.6% of participants (McGuire & O’Higgins Norman, 2017). In another instance, in the EU Kids Online Survey (Livingstone et al., 2011), mothers, as three out of four parents from all the 25 participating countries, were the most aware of children’s Internet use. In another study, 94.1% of participant parents were mothers (Byrne et al., 2014). Therefore, more is known about maternal as compared to paternal awareness, because mothers usually report, particularly when only one parent is required to participate (Symons et al., 2017). This suggests the parental scales be tested for measurement invariance in future research.

Fourth, a measurement invariance test could also be run for children’s age groups. Younger and older children have different needs for Internet use. Parents may, therefore, adjust their mediation strategies as children get older (Staksrud & Ólafsson, 2020). Parents tend to give their adolescents more freedom/space for Internet use (Glatz et al., 2018).

Fifth, the sample size may be considered small, which may account for the low R-squared results. Nevertheless, the predictor variables still account for some variability in the dependent variable. For a higher effect size, future research might consider a bigger sample size and adding other predictor variables related to parent–child-risk characteristics to the model. Additional predictors can increase the explanatory power of the model.

## Implications for Best Practice

Although the findings suggest an intervention program should emphasize restrictive mediation to increase parental awareness, this can be inhibitory to children’s access to online opportunities. Recent evidence from eight European countries indicates that enabling mediation increases the likelihood of meeting children’s needs for safe and purposeful use of the Internet, while restrictive mediation decreases the likelihood of experiencing risks online (Livingstone et al., 2017). As such, a combination of restrictive, enabling, and observant mediation is likely to be the most effective strategy for raising parental awareness.

## Conclusions

The present research was the first to conceptualize and test a unidirectional relationship from parental self-efficacy to parental awareness through parental mediation. The research has hereby proposed a conceptual framework of parental awareness, showing that higher self-efficacy predicts higher awareness through higher restrictive mediation. The framework is hereby expected to enhance understanding of how (a) higher parental self-efficacy directly hinders their awareness and (b) the same parental characteristic indirectly enhances awareness. The novelty of the findings has further manifested itself in the validation and reliability of scales measuring (a) parental self-efficacy, (b) parental mediation, (c) parental awareness, and (d) parental Internet use.
